# A genome-wide scan using tree-based association analysis for candidate loci related to fasting plasma glucose levels

**DOI:** 10.1186/1471-2156-4-S1-S65

**Published:** 2003-12-31

**Authors:** Chien-Hsiun Chen, Chee Jen Chang, Wei-Shiung Yang, Chun-Liang Chen, Cathy SJ Fann

**Affiliations:** 1 The Institute of Biomedical Sciences, Academia Sinica, Taipei, Taiwan; 2 Department of Medical Research, National Taiwan University Hospital, Taipei, Taiwan; 3 Department of Internal Medicine, National Taiwan University Hospital, Taipei, Taiwan; 4 The Graduate Institute of Clinical Medicine, National Taiwan University, Taipei, Taiwan

## Abstract

**Background:**

In the analysis of complex traits such as fasting plasma glucose levels, researchers often adjust the trait for some important covariates before assessing gene susceptibility, and may at times encounter confounding among the covariates and the susceptible genes. Previously, the tree-based method has been employed to accommodate the heterogeneity in complex traits. In this study, we performed a genome-wide screen on fasting glucose levels in the offspring generation of the Framingham Heart Study provided by the Genetic Analysis Workshop 13. We defined one quantitative trait and converted it to a dichotomous trait based on a predetermined cut-off value, and performed association analyses using regression and classification trees for the two traits, respectively. A marker was interpreted as positive if at least one of its alleles exhibited association in both analyses. Our purpose was to identify candidate genes susceptible to fasting glucose levels in the presence of other covariates. The covariates entered in the analysis including sex, body mass index, and lipids (total plasma cholesterol, high density lipoprotein cholesterol, and triglycerides) of the subjects, and those of their parents.

**Results:**

Four out of seven positive regions in chromosomes 1, 2, 6, 11, 16, 18, and 19 from our analyses harbored or were very close to previously reported diabetes related genes or potential candidate genes.

**Conclusion:**

This screen method that employed tree-based association showed promise for identifying candidate loci in the presence of covariates in genome scans for complex traits.

## Background

Problem 1 of Genetic Analysis Workshop 13 (GAW13) provided the data from the Framingham Heart Study. We focused on the offspring cohort due to the missing rate of the data in the parental cohort.

Because the history of medical intervention, including the adjustment of lifestyle and the use of anti-diabetic medications were not available, we chose the highest fasting plasma glucose levels across the course of follow-up as the targeted quantitative trait to indicate the potential risk for abnormal glucose disposal. As suggested by the American Diabetes Association, the impaired fasting glucose (IFG, fasting plasma glucose between 110 and 125 mg/dl) appears as a risk factor for type 2 diabetes mellitus (T2DM) [1]. We further used the lower limit of IFG (≥110 mg/dl) as the cut-off to transform this quantitative trait into a dichotomy. In this way, we included the subjects in the group with one or more incidences of higher fasting plasma glucose. We then performed association analyses using regression and classification trees for the two traits, respectively. A marker was considered positive if at least one of its alleles showed association in both analyses.

Our purpose was to identify candidate genes related to the fasting glucose levels in the presence of covariates. We found a few interesting markers that are closely linked with some potential candidate genes biologically relevant to glucose metabolism.

## Method

### Data processing

For the phenotype measurements, the corresponding covariates were created using their cross-sectional means. The covariates entered in the analysis included sex, body mass index, and lipids (total plasma cholesterol, high density lipoprotein cholesterol, and triglycerides) for each subjects. To control for potential familial correlations, the cross-sectional means of the maternal and paternal phenotype measurements were also included as covariates.

For the genotypic data, an allele was chosen to enter the analyses if its allele frequency is at least 10%. Alleles with frequencies less than 10% but from the same marker are categorized as an incognito allele. The allelic covariates were created using the technique proposed by Zhang and Bonney [2].

### Association analysis using classification trees

The classification tree (CT) and regression tree (RT) methods are both built on the recursive partition technique; they can be used to partition a study population into homogeneous disjointed subgroups. The optimal tree is created by both growing and pruning procedures. The maximal tree is built by splitting each node into two child nodes until the purity of the terminal node is achieved. In splitting, the best choice of the child node is derived while the minimum of the entropy impurity function is reached. In pruning, it is processed for each binary class *j *in the subtree τ until the unconditional misclassification rate  is attained, where c(*j*|*i*) is the cost that a class *j *is classified as a class *i *and *IP *is the entropy impurity function. In general, choice of the cost depends on the severity of the misclassification. In this study, equal cost was chosen for both misclassifications because it frequently gives most satisfactory analyses [3], i.e., c(*1*|*0*) = c(*0*|*1*). The optimal tree in RT is similar to that in CT with a different impurity function , i.e., the within-node variance in the tree τ. More details of CT, RT, and corresponding splitting criteria are described elsewhere [3-5].

Tree-based association analysis was implemented by using genotype measurements such as allelic covariates and related phenotype measurements to construct binary trees. An allele shows association with the trait if its corresponding covariate is included in the optimal tree.

**Figure 1:**
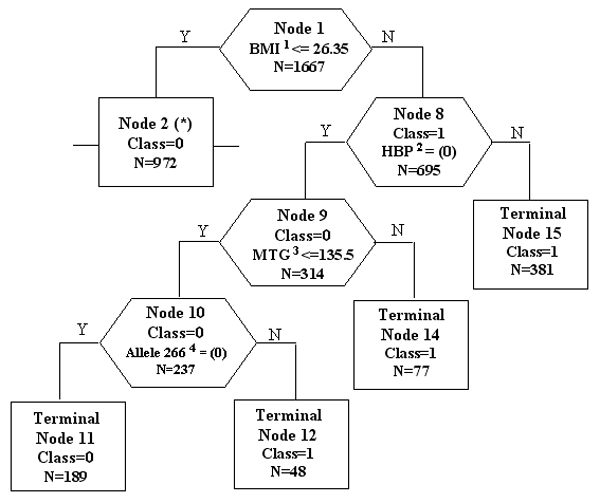
**A proportion of an optimal tree from classification tree **Decision node criteria: 1. BMI: average BMI across-section 2. HBP: ever hypertension in all sections 3. MTG: average maternal triglycerides 4. Allele 266 of D16S2620 Definition of classes: Class 0, glucose levels < 110 mg/dl in all section; Class 1, at least one observed fasting glucose levels >110 mg/dl.

To illustrate the tree construction, a portion of an optimal tree created by CT is presented in Figure 1. First, a total of 1667 subjects (the offspring generation) were divided into two groups according to whether averaged BMI was less than 26.35 or not (node 1 to nodes 2 and 8). Those with averaged BMI higher than 26.35 were further subdivided according to their HBP status (node 8 to nodes 9 and 15). Those 314 subjects in node 9 were further divided into node 10 (or 14) if their averaged maternal triglyceride was lower (or higher) than 135.5 mg/dl. Finally, if the genotype was absent of allele 266 in D16S2620 then the subject was likely to have a fasting glucose levels lower than 110 mg/dl. In summary, allele 266 in D16S2620 was associated with fasting glucose levels for those with higher BMI (>26.35), no HBP, and lower maternal triglyceride levels (<135.5 mg/dl).

### Genome-wide screen

In this study, we conducted a genome-wide screen to identify the candidate gene in the presence of a set of specified covariates. We performed RT- and CT-based association analyses on the quantitative and dichotomy traits, respectively. A marker was interpreted as positive if at least one of its alleles showed association in both association analyses. The allelic covariates from the same chromosome were entered in the analyses simultaneously. The genome-wide screen consisted of 22 such processes for the autosomes. The computer programs QUEST [6] and RT [7] were used to construct the binary trees for the CT and RT analyses.

### Web-searching for candidate genes

The map position was defined using Ensemble Genome Server at Sanger Institute . For candidate gene search, we used Online Medelian Inheritance in Man at National Center for Biotechnology Information  or euGene .

## Results

Table 1 shows these seven candidate regions, consisting of nine positive markers in both analyses, were on chromosomes 1p, 2p, 6q, 11p, 16q, 18p, and 19q. Among these seven regions, four regions, covering the four markers, D1S1665, D6S474, D11S1981, and D19S254, were closely linked to the genes previously reported to be relevant to glucose metabolism or diabetes mellitus (details listed in Table 1).

## Discussions and Conclusions

In this study, the intent of our screen method was to identify candidate markers rather than to pinpoint susceptibility alleles, although it can be applied to detect the allelic or non-allelic heterogeneity. The cut-off value used in CT in this analysis was chosen for a biological reason. However, the analysis was sensitive to the choice of cut-offs when the subjects were largely clustered around the cut-off point (>110 mg/dl). Only three regions on 1p, 16q, and 18p were consistently positive at neighboring cut-offs from 100 to 120.

Although covariates such as BMI and HBP, which are associated with fasting glucose level, were included in our analyses, the cut-off of these covariates in our final 22 optimal trees were not the same. Further studies are needed to inspect the impact of different cut-off and associated alleles.

From a different point of view, our method used the RT analysis on the quantitative trait to validate the results from CT such that the positive markers showed association in both analyses. Notably, four out of the seven candidate regions harbored previously reported genes that are related to glucose metabolism or diabetes mellitus. In conclusion, our screen method shows promise for searching candidate loci in genome scans for complex traits.

## Figures and Tables

**Table 1:** Positive markers found in the analyses using classification and regression trees

Chr	Marker	Marker Alias	Position	Previous Reports
1	D1S1665	GATA61A06	1p31.1	Amylase, salivary, Pancreatic (1p21), facilitated glucose transporter (1p31-35), agiopoietin-like 3 (1p31)
2	D2S2952	GATA116B01	2p25.1	Glucokinase regulatory protein (2p23), Alstrom syndrome (2p13), serum levels of leptin (2p21), hexokinase 2 (2p12), eukaryote translation initiation factor 2-α kinase 3 (Wolcott-Rallison syndrome, 2p21)
6	D6S474	GATA31	6q21	IDDM 1 (6q21) IDDM5 (6q24-27), IDDM8 (6q25-27), IDDM15, transient neonatal diabetes mellitus (6q24), pleomorphic adenoma gene-like 1 (6q24-26), phosphodiesterase 1 (6q22-23)
11	D11S1981	GATA48E02	11p15.1	HRAS (11p14.1), INS (11p14.1), MODY1 (11p15.5), phosphodiesterase 3B (11p15), SUR1 (11p15.1), Kir6.2 (11p15.1)
16	D16S3396	ATA55A11	16q12.1	RRAD (16q22)
	D16S3253	GATA22F09	16q12.2	
	D16S2620	GATA67G11		
18	D18S542	GATA11A06	18p11.21	
19	D19S254	Mfd238	19q13.43	Glycogen synthase 1 (muscle)(19q13.3), ApoCIII (19q13.2), Dystrophia Myotonica 1 (19q13.2-13.3), AKT2 (19q13.1-13.2), TGFβ1 (19q13.1), Glycogen synthase kinase 3A (19q13.1-13.2)
